# The Role of Nitric Oxide in Stem Cell Biology

**DOI:** 10.3390/antiox11030497

**Published:** 2022-03-03

**Authors:** Estefanía Caballano-Infantes, Gladys Margot Cahuana, Francisco Javier Bedoya, Carmen Salguero-Aranda, Juan R. Tejedo

**Affiliations:** 1 Department of Molecular Biology and Biochemical Engineering, Universidad Pablo de Olavide, 41013 Seville, Spain; gmcahmac@upo.es (G.M.C.); fbedber@upo.es (F.J.B.); 2 Department of Regeneration and Cell Therapy, Andalusian Center for Molecular Biology and Regenerative Medicine (CABIMER), University of Pablo de Olavide-University of Seville-CSIC, 41092 Seville, Spain; 3 Biomedical Research Network for Diabetes and Related Metabolic Diseases-CIBERDEM, Instituto de Salud Carlos III, 28029 Madrid, Spain; 4 Department of Pathology, Institute of Biomedicine of Seville (IBiS), Virgen del Rocio University Hospital, CSIC-University of Seville, 41013 Seville, Spain; csalguero-ibis@us.es; 5 Spanish Biomedical Research Network Centre in Oncology-CIBERONC, Instituto de Salud Carlos III, 28029 Madrid, Spain; 6 Department of Normal and Pathological Cytology and Histology, School of Medicine, University of Seville, 41004 Seville, Spain

**Keywords:** nitric oxide, stem cell, evolution, cell signaling, cell differentiation, pluripotency, metabolism, biomaterials, regenerative medicine

## Abstract

Nitric oxide (NO) is a gaseous biomolecule endogenously synthesized with an essential role in embryonic development and several physiological functions, such as regulating mitochondrial respiration and modulation of the immune response. The dual role of NO in embryonic stem cells (ESCs) has been previously reported, preserving pluripotency and cell survival or inducing differentiation with a dose-dependent pattern. In this line, high doses of NO have been used in vitro cultures to induce focused differentiation toward different cell lineages being a key molecule in the regenerative medicine field. Moreover, optimal conditions to promote pluripotency in vitro are essential for their use in advanced therapies. In this sense, the molecular mechanisms underlying stemness regulation by NO have been studied intensively over the current years. Recently, we have reported the role of low NO as a hypoxia-like inducer in pluripotent stem cells (PSCs), which supports using this molecule to maintain pluripotency under normoxic conditions. In this review, we stress the role of NO levels on stem cells (SCs) fate as a new approach for potential cell therapy strategies. Furthermore, we highlight the recent uses of NO in regenerative medicine due to their properties regulating SCs biology.

## 1. Biological Functions of Nitric Oxide

**Figure 1:**
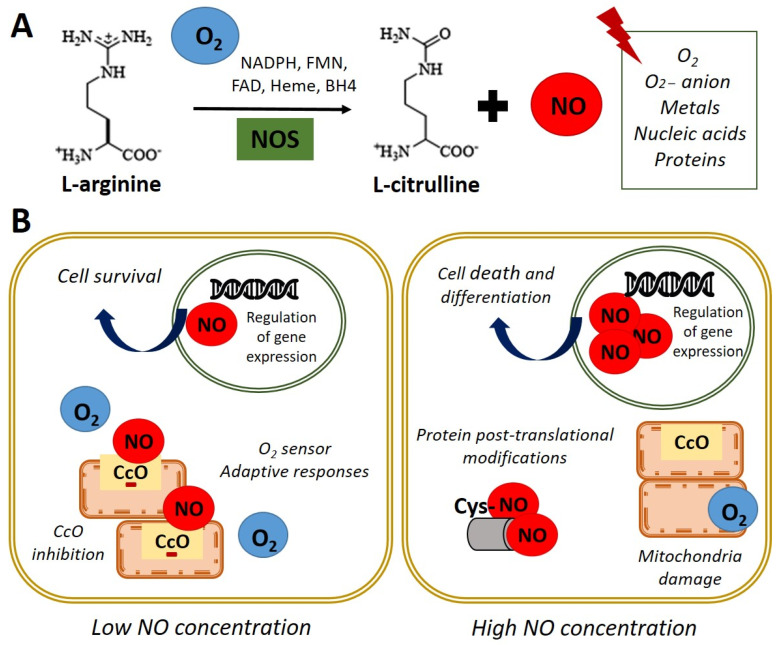
Nitric oxide synthesis and biological functions. (**A**) In normoxia conditions (21% O_2_), nitric oxide synthase (NOS) catalyzes the oxidation of the terminal guanidinyl nitrogen of the amino acid L-arginine to form L-citrulline and nitric oxide (NO) in presence of NADPH and cofactors such as flavin mononucleotide (FMN), flavin adenine dinucleotide (FAD), heme, and tetrahydrobiopterin (BH4) [3]. Once produced, NO readily interacts with O_2_, O^2−^ anion, metals, nucleic acids, and proteins. (**B**) Left panel. NO at low concentration inhibits cytochrome c oxidase (CcO) activity by competing with O_2_. Adaptive responses to O^2^ concentration and cell survival genes are activated. Right panel. High concentrations of NO induce damage in all mitochondrial complexes, nitrosylation, or oxidation of protein thiol groups and induce cell death and differentiation.

Nitric oxide (NO) is a highly reactive gas with a brief life span, synthesized by the enzyme nitric oxide synthase (NOS) through L-arginine oxidation to L-citrulline. In most mammals, the existence of 3 isoforms of the NOS enzyme has been described: NOS1 (nNOS-neuronal), NOS2 (iNOS-inducible), and NOS3 (eNOS-endothelial) [1]. NO is a free radical that interacts with oxygen (O_2_), super-oxide anion (O_2_^−^), metals, nucleic acids, and proteins (Figure 1A). In turn, NO is rapidly oxidized and transformed into nitrate and nitrite in a reversible reaction catalyzed by reductase enzymes activated when endogenous NOS enzymes are dysfunctional [2,3].

NO acts as an essential second messenger involved in numerous biological functions such as regulating blood pressure, through the relaxation of smooth muscle and the inhibition of platelets aggregation, as well as modulating the immune response and as a neurotransmitter in the central nervous system [4]. Furthermore, NO has been shown to affect the expression of genes that regulate cell survival and proliferation, at transcriptional and translational levels, in several cell types [5,6]. Additionally, NO has a role in the pathophysiology of cancer or endocrine and neurodegenerative diseases [7]. Moreover, NO plays a key role in embryonic development, emphasizing its importance in cardiac development and function [8,9].

Regarding the functions performed by NO, it has been reported that these are dependent on their intracellular concentration. On the one hand, physiological concentration modulates cytochrome c oxidase (CcO) activity, depending on the intracellular O_2_ concentration and the redox state of CcO. It was observed in rat lung mitochondria that NO at low concentrations inhibits CcO, competing with O_2_ [10]. This interaction between CcO and NO allows the detection of changes in O_2_ concentrations and the initiation of adaptive responses. On the other hand, high doses of NO can induce post-translational modifications through nitrosylation, or oxidation of protein thiol groups and cause the oxidation of iron (Fe^2+^) of the mitochondrial active centers in rat liver mitochondria, causing damage in all mitochondrial complexes [11]. This evidence indicates that NO could be a physiological regulator of cellular respiration and metabolism (Figure 1B). Furthermore, the potential role of NO in regulating the cellular response to hypoxia in vitro has been described [10,12,13,14]. In these studies, the authors used an interval of oxygen pressure value around 1–5% of O_2_ to induce severe or physiological hypoxic conditions in vitro.

Interestingly, it has been reported the direct effect of NO on processes such as cell proliferation and survival in embryonic stem cells (ESCs) [5]. Besides, increased NO concentrations due to an inflammatory response can cause oxidative effects and nitrosative stress with the consequent induction of apoptosis. These effects are implicated in the pathophysiology of degenerative diseases since they are the cause of chronic cell death [15]. Despite the adverse effects described after the accumulation of high concentrations of NO at the intracellular level, the use of pharmacological treatment with high doses of NO in ESCs has been described to induce differentiation towards certain specific cellular lineages (Figure 1B). Thus, the NO donor molecules are essential components in designing controlled differentiation protocols [6,16,17,18]. This field is intensely discussed in the current review and the recent applications of exogenous NO treatments in regenerative medicine. The summary of the content of the current review on the role of NO in stem cells biology is described in Table 1.

## 2. Nitric Oxide Signaling Pathways in Stem Cell

NO exerts its effects through cyclic guanosine monophosphate (cGMP)-dependent and independent mechanisms [19,20,21]. The independent cGMP pathway is based on NO’s interaction with molecules such as O_2_, anion O_2_^−^ and CcO, among others, due to its high reactivity as previously described [10]. The effect of these reactions is different depending on the concentrations of NO. Nitrosylation and nitration reactions are frequent at high doses of NO [22]. Regarding the cGMP-dependent effects, NO activity is mediated through the soluble receptor guanylate cyclase (sGC), which is a heterodimeric hemoprotein composed of the α subunit (sGCα) and the β subunit (sGCβ) [23]. NO activates sGC by interacting with a heme group catalyzing GTP conversion to cGMP. This activation, in turn, triggers the initiation of a signaling cascade that regulates a wide variety of physiological effects upon interaction with proteins such as the cGMP-dependent protein kinase (PKG) family, cGMP-dependent phosphodiesterases, and cyclic nucleotide-gated channels (CNG) in stem cells (SCs) [20]. Thereafter, it was established that the NO/cGMP route could have an essential role in embryonic development [24] and several additional studies have shown different expressions of NO signaling pathway´s effectors in embryonic cells at various stages of differentiation processes [20,25,26,27].

In this line, NO/cGMP/PKG signaling in bone enhances osteoblasts’ proliferation, differentiation, and cell survival. Thus, sGC is a potential therapeutic target for osteoporosis [28,29,30]. In this context, it has been reported an increase in mouse ESCs (mESCs) differentiation after treatment with iNOS-loaded mineralized nanoparticles that release NO and increase cGMP intracellular concentration, enhancing osteogenesis-related protein levels [31]. Moreover, this pathway is thought to play a crucial role in mediating vasoconstriction, oxidative stress, and fibrosis in cardiomyocytes [32,33]. Accordingly, renovation of adequate NO-sGC–cGMP signaling induced by oral sGC stimulators has been proposed as a noteworthy treatment aim in heart failure [34,35]. Besides, the involvement of NO-signaling in the neuronal differentiation has been assessed. This study revealed that valproic acid (VPA) encourages mature neuronal differentiation of rat adipose tissue-derived SCs through the iNOS–NO–sGC signaling pathway [36]. Additionally, the role of NO enhancing differentiation of mesenchymal SCs (MSCs) to endothelial cells (ECs) has been defined. Bandara et al. reported that NO-producing rat MSCs induce the expression of key endothelial genes while decrease the expression of vascular markers. Moreover, the implantation of a biomaterial scaffold containing NO-producing MSCs enhanced new blood vessels generation, being a novel strategy to vascular regeneration [37].

Alternatively, it has been shown that NO can carry out post-translational modifications such as S-nitrosylation in the cysteine residues of sGC in lung epithelial cells and in Jurkat T cells [38]. In this line, NO’s effects on maintaining pluripotency and differentiation of SCs could also be independent of the sGC-cGMP pathway. In this sense, it was observed an increase in Oct-4 expression in adult mouse bone marrow SCs treated with low dose of NO [39]. However, the mechanisms underlying NO’s effects on SCs stemness are not fully established. Research on NO’s role in SCs fate indicates that this mechanism appears to be independent of the LIF/Stat3 pathway since phosphorylated Stat3 protein levels are not detected in NO-treated ESCs [5]. Moreover, elevated *Nanog* expression from transgene constructs is sufficient for clonal expansion of ESCs, bypassing Stat3 [40]. Chanda et al. showed that genetic or pharmacological blocking of iNOS decreases DNA accessibility and prevents induced pluripotent stem cell (iPSCs) generation. They elucidated that the effect of NO on DNA accessibility is partially modulated by S-nitrosylation of nuclear proteins, as MTA3 (Metastasis Associated 1 Family Member 3), a subunit of NuRD (nucleosome remodeling deacetylase) complex. Furthermore, this study described that overexpression of mutant MTA3, in which the 2 cysteine residues are replaced by alanine residues, impairs the generation of iPSCs from murine embryonic fibroblasts [41].

## 3. Nitric Oxide in the Embryonic Development

As mentioned previously, NO plays an essential role in embryonic development, and it has been described that it can act as a negative regulator of cell proliferation during differentiation and organogenesis in Xenopus and Drosophila [42,43]. Additionally, it has been identified that a critical concentration of NO and cGMP is mandatory for normal mouse embryonic development, and abnormalities from this concentration lead to developmental detention and/or apoptosis of the embryo [44]. This finding claims a key role of NO in embryo development and provides further evidence for the significance of NO generation in murine embryo development and potential in other mammals. Furthermore, NO production is decisive for establishing neural connections in flies, indicating that NO affects the acquisition of differentiated neural tissue [43]. Besides, it was observed that blocking NO production in neural precursor cells in mice resulted in increased cell proliferation [45].

Regarding NO in early development, it is required in cardiomyogenesis processes since it was proven that NOS inhibitors prevent the maturation of cardiomyocytes in ESCs. On the other hand, NOS inhibitors arrest the differentiation of mESCs towards a cardiac phenotype. Thus, NO donors’ treatment can reverse this effect in murine and rat embryos [9]. The expression of the NOS2 and NOS3 isoforms has been reported to occur prominently during the early stages of cardiomyogenesis until it begins to decline around day 14 of embryogenesis [9]. Complementary studies have described that NOS3 expression is increased at early stages and decrease during mESCs differentiation to cardiomyocytes, which suggests that NO could participate in early differentiation events of mESCs and physiological processes in cardiomyocytes [19]. The generation of triply n/i/eNOS (−/−) mice has evidenced the systemic deletion of all three NOSs causes a variety of cardiovascular diseases in mice, demonstrating a critical role of the endogenous NOSs system in maintaining cardiovascular homeostasis [46]. These results constitute substantial support establishing NO’s role as a regulator of organogenesis and embryonic development.

Regarding NO’s role in evolution, reproduction, and fertility, the studies show that this molecule is an essential regulator in ovarian steroidogenesis. NOS is expressed in human granulosa-luteal cells and inhibits estradiol secretion independent of cGMP by straight preventing aromatase activity, downregulation of mRNA levels of the enzyme, and an acute, and direct inhibition of the enzyme activity [47,48]. It has been reported that NO encourages the generation of atrial natriuretic peptide (ANP) and progesterone by human granulosa luteinized cells with minor caspase-3 action hence displaying the role of ANP, progesterone, and NO on the survival of pre-ovulatory human follicle [49]. Additionally, it was described that iNOS and heme oxygenase-1 (HO-1) mRNAs and proteins levels are meaningfully increased in cumulus cells from oocytes not fertilized as compared with the fertilized oocyte. Moreover, not fertilized oocytes showed an increase in NF-κB pathway. These results highlight the role of iNOS and HO-1 as biomarkers of fertility in order to improve human oocyte selection [50]. Furthermore, NO contributes to placental vascular development and function meanwhile the initial phases of pregnancy. Indeed, NO dynamically controls embryo development, implantation, and trophoblast incursion and is one of the key vasodilators in umbilical and placental vessels [51].

## 4. Nitric Oxide and Stemness of Pluripotent Cells

Pluripotent SCs (PSCs) display the ability to proliferate even though preserving the capacity to produce diverse cell types throughout tissue growth and renewal [52]. The molecular mechanisms subjacent these properties are being widely studied, given the potential applications of PSCs in cell therapy. Therefore, the analysis of the mechanisms that regulate the maintenance of pluripotency and differentiation has been the focus of study to establish better differentiation strategies towards specific lineages of clinical interest. In turn, understanding these mechanisms can implement the reprogramming processes towards iPSCs. In this sense, identifying small molecules that act on specific cell signaling pathways that participate in embryonic development provides a useful tool in designing protocols that can efficiently control the “stem state” of SCs and differentiation. Concerning this objective, NO has been described as a molecule used in culture media to promote the differentiation of SCs towards different specific lineages, showing evidence that established its role in the control of tissue differentiation and morphogenesis [6,18]. In general, NO is known for its role as an inducer of apoptosis as was observed in liver cancer cells and in insulin-secreting rat cells [53,54]. Still, it is important to highlight that the induction of apoptosis and differentiation is dose dependent. Its role as a protector of cell death, at low doses, in certain types of cells, as in ESCs or in hepatocytes, has also been described [5,55].

Regarding NO’s role as a differentiation inducer, a study by S. Kanno et al. in 2004 [56] described obtaining cardiomyocytes from ESCs after exposure to high concentrations of chemical NO donors. Subsequently, NO can induce proliferation or differentiation of specific cells, but its effect varies widely depending on each cell type and NO intracellular concentration. For example, the proliferation and differentiation of human skin cells are modulated by NO [57]. In this context, our group has reported that exposure of ESCs to low concentrations (2–20 μM) of the NO donor diethylenetriamine NO adduct (DETA-NO) confers protection from apoptosis; and these effects were also observed in cells overexpressing eNOS. Moreover, this study reported that ESCs exposed to low doses of NO were prevented from the spontaneous differentiation induced by the withdrawal from the cell culture medium of the leukemia inhibitory factor (LIF) in murine and of the basic fibroblast growth factor (bFGF), in human cell lines, respectively. Similarly, we described that constitutive overexpression of eNOS in cells exposed to LIF deprivation maintained the expression of self-renewal markers, whereas the differentiation genes were repressed [5]. Conversely, our group reported that high doses (0.25–1 mM) of DETA-NO induce differentiation of mESCs by repression of *Nanog* and *Oct-4* and promote expression of early endoderm markers [6]. These findings support the dual role of NO in the control of ESCs self-renewal and differentiation.

### 4.1. Nitric Oxide and Stem Cell Differentiation

As previously described, we reported that high concentrations of DETA-NO promote the differentiation of mESCs through the negative regulation of the expression of *Nanog* and *Oct-4*. We showed that *Nanog* repression by NO is dependent on the activation of p53, through covalent modifications such as the phosphorylation of Ser315 and the acetylation of Lys379. In addition, an increase of cells with epithelial morphology and expression of early endoderm markers such as *Pdx1* was revealed. When these cells were exposed to DETA-NO and subsequently, VPA for 6 days, cells with endoderm phenotype and expressing definitive endoderm markers, such as *FoxA2*, *Sox17*, *Hnf1-β* and *GATA4*, were obtained [6]. We then deeper studied the mechanisms involved in *Pdx1* gene regulation by NO in mESCs. We showed that, *Pdx1* expression induced by NO is linked with the release of polycomb repressive complex 2 (PRC2) and the histone acetyl-transferase P300 from its promoter area. These events are accompanied by epigenetic changes in bivalent markers of histones trimethylated (H3K27me3 and H3K4me3), site-specific changes in DNA methylation, and no changes in H3 acetylation [18]. Interestingly, the combined use of small molecules such as DETA-NO, VPA, P300 inhibitor and finally the generation of cell aggregates lead to sequential activation of key signals for pancreatic lineage specification, obtaining cells expressing β-cell markers such as *Pdx1*, *Nkx6.1*, *GcK*, *Kir6.2*, *Glut2*, and *Ins1* that are capable of responding to secretagogues such as high glucose and KCl [18]. This protocol is pioneer in using NO to direct differentiation programs towards pancreatic lineages.

Several studies show NO-mediated induction of apoptosis in different cell types, including pancreatic beta cells [58,59] and hepatocytes [60], among others. However, it has been described that a population of ESCs resists nitrosative stress induced by exposure to high levels of NO, and that they increase the expression of cytoprotective genes, such as heme-oxygenase-1 (Hmox1) and Hsp70. Furthermore, it was reported that these cells resistant to stress-induced by high doses of NO enter a process of cell differentiation [6].

Concerning the role of NO as an antiapoptotic agent, it was reported that this mechanism is orchestrated by Bad phosphorylation through PI3K/Akt-dependent activation in insulin-producing rat cells [61].

Regarding cell differentiation signaling pathways induced by NO, it observed the activation of the phospho-inositide-3 kinase (PI3K)/Akt signaling after exogenous NO treatment. Remarkably, the use of AKT activator in absence of NO did not promote endothelial differentiation of mESCs, suggesting an interdependent association between NO and the Akt activation [62].

### 4.2. Nitric Oxide and Pluripotency

Studies carried out in our laboratory have shown that low doses of DETA-NO (2–20 μmol/L) delay human ESCs (hESCs) differentiation since the addition of NO induces an increase in the expression of *Nanog*, *Oct-4*, and *Sox2*. Furthermore, the expression of the SSEA-4 cell surface antigen characteristic of hESC, which disappears five passages after removal of bFGF, is preserved when the culture medium is supplemented with NO. In addition, it was reported that NO at low doses represses some differentiation markers (*Brachyury*, *Gata4*, *Gata6*, *Fgf5*, *and Fgf8*), which are expressed when bFGF is removed from the culture medium. Similar results were obtained for mESC lines cultured in the absence of LIF and supplemented with NO. This study also reported that constitutive overexpression of NOS3 in cultured cells in the absence of LIF protected these cells from apoptosis and promoted cell survival [5]. Furthemore, it was later described that the culture of mESCs with low dose of NO (2 μM DETA-NO) without LIF in the culture media, promoted substantial modifications in the expression of 16 genes involved in the regulation of the pluripotency state. Additionally, the treatment with DETA-NO induced a high level of binding of active H3K4me3 in the *Oct-4* and *Nanog* promoters and H3K9me3 and H3k27me3 in the *Brachyury* promoter. Moreover, it was observed the activation of pluripotency pathways, such as Gsk3-β/β-catenin, in addition to PI3K/AKT activation, which supports the protective effect of NO at low doses against apoptosis. Finally, mESC proliferation decreased, coinciding with the arrest of the cell cycle in the G2/M phase [63]. These results suggested that NO was necessary but not sufficient for maintaining pluripotency and preventing cell differentiation of mESCs.

Numerous studies have reported that NO prevents apoptosis in mESCs by regulating the expression of proteins of the Bcl2 family [5,64]. Increased expression of *Bcl2* has been observed in long-term cultured mESCs cultures in an undifferentiated state that maintains their differentiation potential [65]. In the same line, overexpression of the porcine *BCL2* gene significantly promotes porcine iPSCs survival without compromising their pluripotency [66].

At low levels, NO provides resistance to tumor necrosis factor α (TNF-α) produced by hepatotoxicity in rat hepatocytes [67] and inhibits apoptosis in B lymphocytes induced by Fas [68]. Similarly, it has been reported that low concentrations of NO protect bone marrow stromal cells (BMSCs) from spontaneous apoptosis [69].

In studies carried out by our group, it has been shown that low levels of NO-induced NOS3 overexpression increase the survival of pancreatic beta cells through IGF-1 activation and insulin-induced survival pathways [16]. Our group has also described that exposure to DETA-NO (2–20 μmol/L) protects ESCs from apoptosis through processes that include the decrease of Caspase-3, combined with the degradation of poly (ADP-ribose) polymerase. A reduction in the expression of pro-apoptotic genes, Casp7, Casp9, Bax, and Bak1; and an increase in anti-apoptotic genes, such as Bcl-2 and Birc6 was observed [5]. Additionally, the genomic studies present evidence about the regulation of apoptosis, survival, and response to hypoxia by low doses of NO in mESC. These studies showed the repression of genes involved in the degradation of hypoxia-inducible factor, HIF-1α, the main regulator of the response to hypoxia, together with the overexpression of genes involved in glycolytic metabolism [63]. Considering these preliminary results, we proposed low doses of NO under normoxic conditions could activate a response similar to hypoxia; and that this activation may be responsible for the mechanism by which NO promotes pluripotency and delays differentiation into ESCs and iPSCs. In this line, we recently described the essential role of NO inducing a cellular response to hypoxia in PSCs [14].

## 5. The Role of Nitric Oxide in Metabolic Signature in Stem Cells: Hypoxia-like Response Encouraged by Low Nitric Oxide

**Figure 2:**
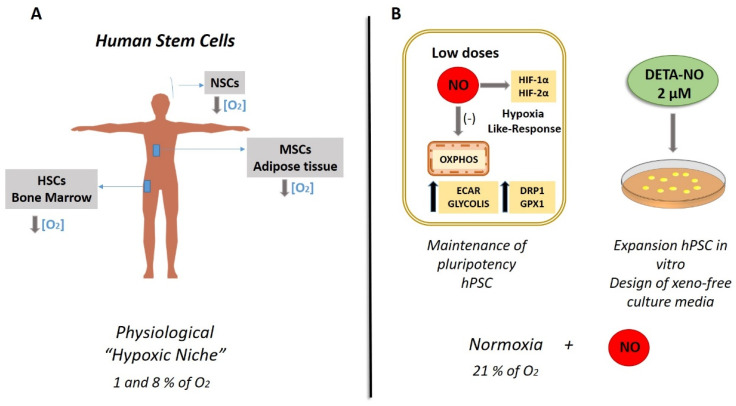
Physiological human stem cell niches versus nitric oxide-based in vitro cultures. (**A**) Hypoxic niches of stem cells (SCs). The figure shows the O_2_ tensions characteristic of the biological niches of MSCs, NSCs, and HSCs. Various studies have determined by direct measurement or applying various mathematical models the O_2_ tension in bone marrow, adipose tissue, and the ventricular zone of the brain where the respective SCs reside. The results showed levels between 1 and 8% of O_2_, revealing an O_2_ tension lower than the atmospheric one (21% of O_2_). Direct measurements in the brain have not been carried out, but tensions of 0.55% O_2_ have been recorded in other attached regions in rodents, and conceptual translation to humans has been carried out. MSCs (mesenchymal SCs); NSCs (neural SCs); HSCs (hematopoietic SCs) [76]. (**B**) Low DETA-NO in normoxia (21% O_2_) encourages a response similar to hypoxia in PSCs. Therefore, exposure to 2 µM DETA-NO led to the accumulation of HIF-1α and HF-2α proteins, increased expression of pluripotency genes *NANOG* and *OCT-4*, and a shift towards the expression of genes favoring glycolitic metabolism. Mitochondrial functions and dynamics were also affected, with a decrease in OXPHOS, an increase in ECAR and *DRP1* expression. These actions are not dependent on changes in mROS levels while GPX1 mRNA expression increase, which is compatible with the maintenance of pluripotency as it is described in the review. All in all, these outcomes indicate that NO induces a hypoxia-like response, regulating mitochondrial functionality and metabolic parameters in human PSCs, thus maintaining pluripotency [14]. In this context, the use of NO is proposed to supplement culture media for human PSCs (hPSCs) expansion. NO (nitric oxide); OCR (O_2_ consumption rate); ECAR (extracellular acidification rate), OXPHOS (oxidative phosphorylation).

The molecular mechanisms by which cells respond to low O_2_ availability (hypoxia) are evolutionarily highly conserved and involve a sequence of changes that regulate gene expression to maintain O_2_ homeostasis and tissue reoxygenation and promote remodeling energy metabolism to maintain survival. Specifically, the O_2_ levels of the microenvironment, or biological niche, play a crucial role in the self-renewal and differentiation of SCs, and are essential for the function of these cells. In this sense, one of the characteristics of this biological niche of MSCs is the low O_2_ tension, which is why it is called a “hypoxic niche”, as is the case of hematopoietic SCs (HSCs) (Figure 2A) [70]. The regulation of the response to hypoxia is directed mainly by the transcriptional activity of Hypoxia-inducible factors (HIFs) [71]. Recent studies have shown evidence of the importance of cultures in hypoxia to favor the process of cell reprogramming and promote pluripotency by maintaining stable levels of HIFs [72,73,74,75].

Regarding SCs niche, a low concentration of O_2_ is a physiological feature that supports pluripotency maintenance [77,78]. Under this condition, PSCs show a metabolic adaptation where glycolysis is increased [79,80]. Thus, previous studies have stated that hypoxia can increase the survival of neural crest SCs [69] and HSCs [70] and can prevent the differentiation of ESCs [75]. In this context, it has been reported that hypoxic conditions (3% O_2_) can increase the reprogramming effectiveness of mononuclear cells from peripheral blood after nucleofection by episomal vectors [81]. Besides, hypoxic atmospheres are considered suitable for inducing differentiation of PSCs [82,83,84,85,86].

Concerning NO and SCs fate, we reported that low NO levels contribute to the preservation of stemness in mESCs by controlling pluripotency genes, energy metabolism, and mitochondrial function, as we mentioned previously [63]. Several studies have described NO’s effect on HIF-1α accumulation and metabolic profile in cancer and somatic cells [87,88,89,90,91,92]. Moncada et al. reported that a high dose of NO triggers HIF-1α steady levels by an independent mitochondria pathway in human cancer cell line [12]. Moreover, they also revealed that HIF-1α accumulation induced by exogenous NO was not reliant on O_2_ concentration. In this line, for the first time, our group studied the effect of low NO on the hypoxia response in human PSCs (hPSCs). We found that the treatment with low NO doses promotes HIF-1α and HIF-2α protein accumulation in hPSCs cultured under normoxic conditions (21% O_2_), mimicking the hypoxia response in cells cultured at 5% of O_2_ in a hypoxia incubator [14] (Figure 2B). We also revealed an increase in the expression of *NANOG* and *OCT-4* mRNA and protein levels in human iPSCs exposed to a low dose of DETA-NO in normoxia when compared with untreated cells. Likewise, it has been described that HIF-2α triggers Oct-4 mRNA expression levels, a key gene of pluripotency [94], and HIF-1α and HIF-2α are mandatory in the initial phases of reprogramming of somatic cells, orchestrating metabolic changes to reach the undifferentiated state [72,93]. Additionally, it has been stated that overexpression of HIF-1α and HIF-2α permits the metabolic modification required for cellular reprogramming, with enhanced expression of glycolytic genes [72,93].

Concerning NO’s role in metabolic regulation, we reported an increase in glycolytic genes (*HK2*, *LDHA*, *PDK1*, and *PKM2*) and angiogenesis gene, vascular endothelial growth factor A (*VEGF-A*), in hPSCs after low dose of DETA-NO treatment, showing comparable results in cells grown under hypoxia conditions (5% of O_2_) [14]. These results are in line with those reporting a relevant role for anaerobic glycolysis in preserving cell stemness, a cell fate that is endorsed by upregulation of glycolytic gene as GLUT3 and PKM2 [94,95,96]. Formerly, it has been reported that low doses of NO encourage a reversible inhibition of mitochondrial CcO, decreasing O_2_ consumption rate and ATP generation throughout a block of electron flux at Complex IV in somatic and cancer cells [97]. We also observed that low NO downregulates oxidative phosphorylation (OXPHOS) and increases mitochondrial fission by increasing dynamin-related protein 1 (*DRP1*) and a decrease in mitofusin2 *(MFN2*) expression, a key mitochondrial fusion gene, in hPSCs cultured under normoxia (21% O_2_) conditions. Furthermore, we described for the first time that low NO declines oxygen consumption rate (OCR) and rise extracellular acidification rate (ECAR) in hPSCs [14]. Concerning mitochondrial dynamic, the fission protein *DRP1* plays a crucial role in preserving iPSCs colonies [94,98]. Upregulation of *DRP1* expression and activation are linked with low mitochondrial activity, a key feature of metabolic profile of SCs; hence, this outcome supports NO’s role as an inducer of pluripotency in hPSCs [96]. Regarding the role of *MNF 2* in stemness, it has been stated that HIF-1α knockdown significantly increases MFN2-mediated Wnt/β-catenin signaling, and extreme mitochondrial fusion could also promote the neural SCs differentiation potential of hiPSCs via triggering the β-catenin signaling. This evidence supports that the block of HIF-1α decreases the pluripotency and self-renewal potential of hiPSCs [99].

In line with these results, we described that low NO decreases mitochondrial activity, but we did not appreciate a significant increase in mROS (mitochondrial reactive oxygen species). However, an increase in the antioxidant enzyme glutathione peroxidase-1 (GPx-1) expression was observed as a homeostatic response (Figure 2B) [14]. In this context, it has been previously reported that the expression level of GPx-1, is intensely reduced upon the differentiation. Moreover, genetic and pharmacology inhibition of GPx-1 expression enhances SCs differentiation. In this study, these authors showed fast degradation of GPx-1 throughout early differentiation, highlighting the critical role of GPx-1 in self-renewal regulation in mESCs [100]. In this context, these outcomes support NO’s role as a protector against oxidative stress delaying spontaneous differentiation proceedings.

Thus, NO’s role as a hypoxia-like response inducer has been stated, but the underlying molecular mechanism has not been completely clarified. In this sense, our group observed that the treatment with low NO significantly decreases P402 hydroxylation in cells cultured under normoxia (21% O_2_) [14]. According to this result, it has been stated that NO steadies HIF-1α by S-nitrosylation of thiol groups and likewise by the straight block of prolyl hydroxylase domain proteins (PHDs), preventing HIFs degradation [101,102]. In our study, we similarly found decreased proteasomal activity in NO-treated PSCs [14]. In this regard, it was described that NO reduces proteasomal degradation through S-nitrosylation or O-Glc-N-acetylation observed in normal rat kidney epithelial cells (NRK line) and in rat aortic smooth muscle cells [103,104]. Besides, NO is involved in protein homeostasis by S-nitrosylations of HSPA8 and the ubiquitin ligase UBE2D in neuronal cells. Direct S-nitrosylation of UBE2D reduces ubiquitination and proteasomal degradation [105].

Our results point out that the regulation of stemness by NO hinge on HIF1-α balance and activation of the biological response to hypoxia, by the regulation of energy metabolism, mitochondrial function, and the preservation of pluripotency in hPSCs. Concerning NO’s role as a hypoxia mimetic, disagreement occurs as to whether it induces HIF-1 α accumulation [87,101] or HIF- 1α degradation [12], but these studies always reference high doses of NO, encouraging SC differentiation and apoptosis. However, we have proposed low NO promotes HIF-1α stabilization under normoxic conditions (Figure 2B). Hence, our results highlight the double action of NO in stemness, increasing cell survival and promoting pluripotency at low NO, or as an inducer of differentiation at higher concentrations [6]. Because of this knowledge, from a biotechnological perspective, low NO might be used as a replacement for hypoxic conditions in large-scale expansion of PSCs needed for cell therapy avoiding the high economic burden of hypoxia incubators [14].

## 6. Exogenous Nitric Oxide in Regenerative Medicine

Due to NO’s biological role in SCs biology it has been widely used as a pharmacological molecule in the regenerative medicine field. The dual role of NO on cell fate of PSCs, makes this molecule can be used for regenerative medicine proposal in two senses: (i) as a differentiation inducer and (ii) to preserve stemness, as it has been previously discussed. Considering the different dose-dependent effects of NO, it is mandatory to adjust the accurate concentration to use this molecule in regenerative medicine. In this line, the use of biomaterials to control NO doses delivery in situ has been implemented due to their benefits in tissue regeneration and cell therapy [106]. Current studies have improved local adherence and viability after SCs transplant using biomaterials [107]. Regeneration level and immunomodulation have increased after local combinations of NO donors and bio-nanomaterials, conferring optimal conditions for tissue repair [108]. Several strategies have been designed to release NO via catalytic or noncatalytic methods. Many NO donors (DETA-NO, GSNO, SNAP, etc.), natural enzymes, and enzyme mimics are highlighted, and recent promising developments of NO-releasing scaffolds, nanoparticles, and films are presented. In this line, there are in the literature several strategies engineered as [106]:

- NO distribution from small molecule NO donors
- NO transfer from injectable materials:
  *Liposomes*
  *Micelles*
  *Dendrimers*
  *Silica and gold nanoparticles*
  *Polymer particles*
  *Metal–organic frameworks*
- NO delivery from implantable materials
- Localized synthesis of NO using natural enzymes and synthetic prodrugs
- Enzyme mimics for conversion of endogenous prodrugs of NO

These biotechnological tools have been designed to control NO release, managing physic-chemical parameters as NO payload, maximum NO flux, NO release half-life, the period required to reach maximum flux, and interval of NO release [106].

Regarding the biotechnological applications of NO in the regenerative medicine field, we found exciting results more than two decades ago. Shabani et al. developed polyethyleneimine cellulose NONOate polymer to release NO in a controlled manner in aqueous media with a half-life of 16 h. This polymer was designed to treat rats’ dermal wound, enhancing wound healing without toxic effects [109]. In this line, recently, a new polymer, polyethylenimine-based diazeniumdiolate NO donor, designed to deliver NO in a controlled way, showed positive results in cutaneous healing with a high grade of biocompatibility [110]. In addition, it has been reported that VEGF release is modulated by NO over angiogenesis taking place in bone remodeling [111]. Thus, the use of NO for the traumatic orthopedic regenerative medicine has been currently proposed [112]. It is also known that NO derived from eNOS plays a key role in bone formation observed through eNOS knockout mice models [113]. Furthermore, NO manages main processes for tissue regeneration at moderate levels, including collagensynthesis, proliferation, and cell differentiation [114]. The recent and essential progress in the effects of NO donors in bone tissue highlights the promising design and use of NO donors allied to biomaterials in the sustained and localized NO release for bone tissue regeneration.

Concerning the role of NO in SCs biology, previously, the effect of NO scaffolds on osteogenic differentiation of human mesenchymal bone marrow cells has been presented [115]. The proper concentration of NO in situ is essential during a prolonged period to obtain the benefits on osteoblast function [30,112]. In this line, 3D bone scaffold designed for nanoparticulated NO release has shown benefit as an antimicrobial device and tissue regeneration, improving fracture healing through enhancing angiogenesis and osteogenic differentiation [116]. These results point out NO as a molecule of interest to improve fracture healing. Its roles in fracture-site decontamination, mediating inflammation, endorsing angiogenesis, and bone tissue renovation could allow numerous intervention points within the fracture healing cascade.

Regarding cell signaling induced orchestrated by NO, activation of JNK/MAPK pathway modulating osteoblast and adipocyte lineage differentiation in periodontal ligament SCs. In this line, NO is essential for maintaining the balance between osteoblasts and adipocytes in human periodontal ligament SCs via the JNK/MAPK signaling pathway [117].

In the odontology field, an in vivo study in dogs confirmed that NO-releasing biomimetic nanomatrix gel endorsed tooth neovascularization with the growth of root canals. This kind of bio-gel that permits a controlled release of NO with an optimal concentration has been recommended for human trials due to its potential as a root treatment material for tissue renewal [118]. In this context, a current study described that rat dental pulp SCs (rDPSCs) encouraged by exogenous NO (NOC-18) might differentiate into odontoblast-like cells with enhanced alkaline phosphatase (ALP) activity and expression levels of odontoblast-specific genes such as runt related factor 2 (Runx2), DMP 1, and dentin sialophosphoprotein through the NF-κB pathway, highlighting promising therapeutic possibilities for NO treatment in the odontology tissue [119].

Besides, NO significantly promotes angiogenesis and develops mature blood vessels via recruiting perivascular and ECs [120]. Consequently, the use of exogenous NO in cardiovascular regeneration is very remarkable. In this context, enzyme-functionalized vascular grafts that catalyze in-situ discharge of NO, directed and locally, from exogenous NO prodrug are promising strategies in cardiovascular tissue regeneration. A recent study shows replacement of thrombus development in vivo and enhancement of vascular tissue regeneration and renovation on the grafts in rat abdominal aorta demonstrating that this method may be beneficial to develop new cell-free vascular implants for management of vascular diseases [121]. Interestingly, Kabirian F et al., showed the NO-releasing small-diameter vascular grafts (SDVGs) dramatically enhanced ECs proliferation and significantly improved ECs migration in-vitro, in Human Umbilical Vein Endothelial Cells (HUVEC) compared to control grafts. In addition, the NO-releasing SDVGs showed neovascularization potential in-vivo, in a chick chorioallantoic membrane. These findings are expected to facilitate endothelium regeneration and integration of personalized vascular implants with enhanced clinical success [122]. The use of biodegradable scaffolds that induce endothelium regeneration, are also promising therapeutically strategies for improving performance of SDVGs. A recent study has reported microfibrous polycaprolactone (PCL)/gelatin scaffolds synthetized by electrospinning and activated with heparin and organoselenium-immobilized polyethyleneimine for NO production through layer-by-layer self-assembly. Results showed that the new scaffolds increase NO generation and improve the attachment of ECs, showing high rated of hemocombatility, and smooth muscle cell regeneration [52]. In the same way, other in vivo study, in adult New Zealand White Rabbits (2.5–3.5 kg), has described the development of an endothelium-biomimetic coating linked with heparin and discharging pharmacological doses of NO for bioengineering of cardiovascular stents [123]. Furthermore, it has been designed a nitrate-functionalized patch, in which a biodegradable polymer locally releases NO when is implanted onto the ischemic myocardium. The results showed mitochondrial protection and improved cardiac repair. Regarding mitochondria, they found a decrease in mitochondrial function of complexes I and II by s-nitrosylation of mitochondrial proteins after NO patch implantation. Thus, hydrogen peroxide (H_2_O_2_) level was reduced in the infarcted tissue. About post translational modification induced by NO, s-nytrosylation has been previously reported in mitochondria, thus blocking complexes activity, and reducing ROS accumulation in cardiac tissue [124]. These authors found a reduction in ROS level that prevent cell apoptosis and improve cardiac damage. Moreover, this kind of biomaterial showed efficacy both in mouse and porcine model of myocardial infarction. All in all, these results support the application of NO´s patch for managing ischemic heart injury [125]. Mechanistically, it has been described an increase in LOX (Lysyl Oxidase) functional activity within 3D aneurysmal smooth muscle cells cultures in GSNO (S-nitrosoglutathione) presence and significantly higher deposition of fibrillar forms of elastic matrix-associated proteins such as fibrillin-1, and fibulins-4, 5, which is highly hopeful for therapeutic tissue engineering and in situ elastin regeneration [126]. Additionally, it has been described a role of NO as hESCs-derived cardiomyocytes protector against Ischemia/Reperfusion (I/R) induced damage. Interestingly, NO-donor SNAP activates sGC in a model of simulated ischemia. The NO-donor S-nitroso-N-acetylpenicillamine, SNAP (10^−6^, 10^−5^ M) significantly reduced cell death in differentiated SCs, so stress their cytoprotective effect [127]. According to our results about low NO and induction of a similar hypoxia response in PSCs, mentioned above [14], the treatment with low doses of NO could improve cell survival of cardiomyocytes inducing an adaptation to ischemic atmosphere.

In this sense, aerobic exercise has been described as a beneficial intervention to improve cardiovascular regeneration. About molecular mechanism underlying the positive impact of physical training in heart injuries it was found an increase in NO generation in cardiac tissue that support the maintenance of cardiovascular homeostasis. NO induces cellular pathway that protect SCs against hostile environment as hypoxia condition in the damaged tissue. In this line, it has been previously described that improvement in the adaptation of SCs to hypoxia/reoxygenation environment increases graft SCs survival in ischemic mouse heart [128]. Due to, SCs preconditioning by and local enhancing of NO signaling can be proposed as promising methodology to improve the post-transplantation SCs survival and the effectiveness of cardiac SCs therapy [129]. Agreeing with our results reported, the treatment with low doses of NO induces a hypoxia-like response in PSCs in cells cultured in vitro that could help to increase cell survival after transplantation in a hypoxic tissue [14].

Currently, the locally delivery of NO in a controlled manner during a prolong period is still a challenge. Yang et al. [130] developed a method to provide glutathione peroxidase (GPx)-like activity in the network of copper-phenolic-amine to enhance NO production capacity. The resultant NO-generating coatings, which were fabricated using a natural plant polyphenol, gallic acid, a glutathione peroxidase-like species selenocystamine, and the Cu^II^ ion, exhibited long-term, stable, and controllable NO generation. Since anti-thrombogenicity is a crucial factor for the long-term success of vascular implants, surface heparinization was further introduced through a stepwise metal (copper)-catechol-(amine) (MCA) surface chemistry strategy. The heparin-grafted and NO-catalytic coating could mimic the physiological functions of native endothelium, which may address the challenges of in-stent restenosis [131].

Among new strategies to use NO in SCs therapy it has been developed a method to induce endothelial differentiation of mESCs using a controllable chitosan NO-releasing hydrogel. ESCs were cultured onto the hydrogel system, and the expressions of endothelial differentiation markers were upregulated after NO treatment. Considering these findings, it was confirmed that NO treatment during a continuous period and controlling doses manner is a simple and efficient approach for inducing the endothelial differentiation of ESCs without using growth factors [62].

Currently, it has been reported the development of a direct and safe NO delivery strategy based on layer-by-layer assembled nanocoating to the surface of human vascular ECs as a means to overcome the limitations of conventional NO delivery systems to target cells due to the limited diffusion distance and life of NO. Additionally, VEGF was conjugated to VEGFR of ECs surface to enhance cellular functions, such as cell survival, migration, and angiogenesis along with NO. Results suggested that this technique on nanocoating-based NO and VEGF co-localization to cells can be beneficial for vascular development in the field of SCs transplantation and organoid platform [131]. A similar study has showed that the encapsulation of the NO-donor S-nitrosoglutathione (GSNO) into chitosan nanoparticles (CS NPs) overcame NO drawbacks in pharmacological applications, such as, its short half-life. The ability of GSNO-CS NPs, combined with UV irradiation, to deliver NO was evaluated using ex vivo human skin. The results showed that the combined treatment significantly increased the NO and S-nitrosothiol levels in human skin samples. This effect can reproduce the cardiovascular benefits without negative side effects of skin exposure to UV light [132]. In the same way, a recent study has described the development of an in-situ forming Fmoc-dipheylalanine hydrogel releasing s-nitroso-n-acetylpenicillamine (FmocFF-SNAP) for mice renal (I/R) injury. Fmoc-FF hydrogel comprising of β-sheet nanofibers was prepared through the pH-titration method. Moreover, they measured the expression NOS2 and NOS3 to assess the therapeutic efficiency in the mice renal I/R injury model. Results showed that mocFF-SNAP exhibited a sustained NO release over 7 days in a concentration-dependent manner and caused superior recovery compared to free SNAP in the mice renal I/R injury model [133]. Interestingly, an additional study has reported the design of a new keratin-based NO donor termed S-nitrosated keratin (KSNO), combined with polyurethanes (PU) and gelatin (Gel) to generate PU/Gel/KSNO biocomposite mats. They showed that the PU/Gel/KSNO mats could sustain NO release for a 36-h period without cell toxicity and promote cell proliferation and adhesion of murine fibroblasts and HUVECs, accelerating wound healing in rats without inflammatory reaction in ECs [134].

Lastly, it has been elucidated a novel role of NO in reprogramming MSCs towards an endothelial lineage. Concretely, they establish an endothelial-specific eNOS-NO signaling pathway in rat bone marrow-derived mesenchymal stem cells (BMSCs) via lentiviral vector expression of eNOS and a mutated (F92A) caveolin-1 gene and to evaluate its effect on promoting endothelial differentiation. Results demonstrated that increased level of NO and CAV-1F92A interaction can induce endothelial differentiation via activation of the downstream Wnt/β-catenin signaling pathway. Moreover, it was showed that subcutaneous implantation of NO-producing MSCs seeded in a biomaterial scaffold (NovoSorb^®^) resulted in the survival of transplanted cells and the formation of blood vessels in nude rats [38].

**Figure 3:**
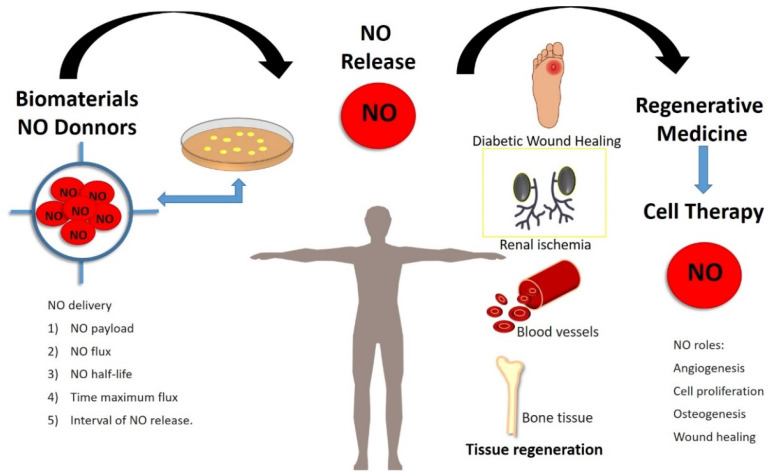
Exogenous nitric oxide (NO) treatment in regenerative medicine. Recently, several strategies to release NO in a controlled manner have been designed to improve NO’s benefits in tissue repair derivate their physiological roles in stem cell biology. Significant advances in the tissue bioengineered field have been stated, highlighting NO roles in bone regeneration, cardiovascular repair, and wound healing.

All these findings together support the use of exogenous NO in combination with devices/biomaterials in regenerative medicine (Figure 3).

## 7. Conclusions and Prospects

In the current literature review, we have highlighted the effects of NO in SCs biology. Several studies have focused on NO’s role in regulating embryo development and organogenesis and many physiological functions such as metabolism and pluripotency in SCs, due to their therapeutic applications. Both, NO-GMPc signaling and post-translational modification orchestrated by NO, as s-nytrosilation, play an important role in pluripotency and cell differentiation in SCs. In this line, it has been reported the dual role of NO in SCs fate. Thus, low NO promotes the maintenance of pluripotency and high doses of NO induces apoptosis and cell differentiation. In PSCs, mitochondrial function and cell metabolism are related to the control of cell stemness fate. In this context, low NO reduces mitochondrial activity and enhances anaerobic metabolism. This metabolic signature is compatible with the maintenance of the undifferentiated state of SCs. In this review, we emphasize NO’s role as a hypoxia-like inducer in human PSCs by stabilizing HIFs under normoxia conditions (21% O_2_) preventing spontaneous differentiation. Subsequently, NO is proposed as an excellent candidate to supplement culture media to improve PSCs expansion without hypoxic atmospheres. Regarding NO applications in regenerative medicine, this review highlights the recent advances in NO´s role in tissue repair, NO´s bioavailability and potential NO-releasing scaffolds, thus obtaining outstanding results in bone repair, wound healing, and cardiovascular regeneration.

## Figures and Tables

**Table 1:** The role of NO in stem cells biology.

Sections	Main Findings	References
Section 1. Biological Functions of Nitric Oxide	NO’s biosynthesis NO’s functions in cells	[1,2,3,4,5,6,7,8,9,10,11,12,13,14,15,16,17,18]
Section 2. Nitric Oxide Signaling Pathways in Stem Cell	Molecular mechanism underlying NO role in stem cell *NO-cGMP pathway* *NO and posttranslational modifications*	[19,20,21,22,23,24,25,26,27,28,29,30,31,32,33,34,35,36,37,38,39,40,41]
Section 3. Nitric Oxide in the Embryonic Development	NO in embryogenesis NO role in oocytes maturation	[42,43,44,45,46,47,48,49,50,51]
Section 4. Nitric Oxide and Stemness of Pluripotent Cells Section 4.1. Nitric Oxide and Stem Cell Differentiation Section 4.2. Nitric Oxide and Pluripotency	The dual role of NO in stemness. *High NO doses induce cell differentiation* *Low NO doses maintain pluripotency*	[5,6,14,16,52,53,54,55,56,57,58,59,60,61,62,63,64,65,66,67,68,69]
Section 5. The Role of Nitric Oxide in Metabolic Signature in Stem Cells: Hypoxia-like Response Encouraged by Low Nitric Oxide	NO as a hypoxic mimetic in stem cells under physiological conditions	[14,70,71,72,73,74,75,76,77,78,79,80,81,82,83,84,85,86,87,88,89,90,91,92,93,94,95,96,97,98,99,100,101,102,103,104,105]
Section 6. Exogenous Nitric Oxide in Regenerative Medicine	Recent advances in NO applications in the tissue bioengineered field: wound healing, bone regeneration and cardiovascular disease. Novel biomaterials to control delivery of NO in situ	[106,107,108,109,110,111,112,113,114,115,116,117,118,119,120,121,122,123,124,125,126,127,128,129,130,131,132,133,134]

Summary of the content of the review on the role of NO in stem cells biology.
